# Study of the influence of socio-economic factors in the international expansion of Spanish franchisors to Latin American countries

**DOI:** 10.1371/journal.pone.0190391

**Published:** 2018-01-02

**Authors:** José M. Ramírez-Hurtado, Juan M. Berbel-Pineda, Beatriz Palacios-Florencio

**Affiliations:** 1 Department of Economics, Quantitative Methods and Economic History, Pablo de Olavide University, Seville, Spain; 2 Department of Management and Marketing, Pablo de Olavide University, Seville, Spain; Public Library of Science, UNITED KINGDOM

## Abstract

The saturation of the domestic market is one of the factors which drive firms to expand their business to other markets. Franchising is one of the formats adopted by companies when establishing their internationalization strategy. Spain is a country where franchising is strongly consolidated. This degree of maturity means that many chains seek other countries in which to operate. This work’s specific aims are, on the one hand, to offer a general view of the current situation of Spanish franchisors in Latin American countries and, on the other hand, to analyze which the socio-economic or external factors are that determine the presence of Spanish franchisors in this market. Canonical-correlation analysis is used to do so. The results show that Spanish franchisors focus on the market’s potential and size, and the per capita income, while they do not take into account its unemployment level, the country risk or the competitiveness there. This work shows that there is a series of socio-economic factors which influence the final choice of the destination country. However, this decision is not solely based on this country’s socio-economic aspects, but also on the structure of the franchising firm itself and on its export experience in other markets. This study therefore complements other research and helps franchisors in their difficult decision of choosing the destination for their internationalization.

## Introduction

Globalization and emerging markets create huge internationalization opportunities for domestic franchisors [1]. Franchising is a promising and increasingly used organizational form to improve strategic, organizational, professional and client-related results in many sectors [2]. Franchising is a business format which provides firms with an excellent opportunity to expand their business to international markets [3]. The saturation of the domestic markets, especially in the developed countries, has forced firms to seek new markets [4]. When establishing their internationalization strategy, firms can choose among different forms—direct investments, collaboration agreements, or franchising, among others. Franchising is one of the international expansion strategies most preferred by many firms [5]. Franchising is a growth model for international franchisors to expand abroad that involves minimal financial risk and a quick go-to-market strategy [6]. The attractiveness of franchising for international expansion is mainly due to its requiring a low level of capital investment and there being an adequate control of its distribution processes [7].

Significant changes have taken place in recent decades in the economies of many industrialized countries through firms opening to new markets. This unstoppable process of internationalization has not only been carried out by large operators but also by medium and small retailers [8]. Although the international franchising industry has become a major player in the development of countries, it has been uneven [9].

The globalization prevailing nowadays means that there is an intensification of trade in local markets and greater levels of competiveness. This causes firms to have to seek new markets in order to be able to survive.

In the specific case of Spain, joining the community project of the European Union has had an effect on the growing external Spanish business fabric. This increase in the number of Spanish firms in European markets has provided these firms with important experience. This has contributed to their presence in other markets, apart from those of Europe, such as those in the American and Asian continents, among others. In this way, the number of Spanish firms in the international markets of goods and services has progressively increased. Indeed, the international dimension has become a relevant part of these firms’ strategies.

In the specific case of franchising, Spanish franchisors have used internationalization as a strategic growth option and as one of the best ways to increase the recognition and renown of their trade names and brands [10]. The current situation of the economic crisis, which has existed in Spain for various years, has meant that the seeking of business opportunities in other countries has accelerated.

According to the 2015 report of the Spanish Association of Franchisors [11], the figures of franchising in Spain shows that there were 1,199 trade names at the end of 2014, which had a total of 63,869 outlets of which 19,250 were own outlets and 44,619 franchised outlets. According to these data, franchising generated 159,822 jobs through franchised outlets and 89,092 jobs via own outlets in 2014. In the same year, franchising billed more than 25,879 million euros. The aforementioned figures show that franchising is strongly consolidated in Spain, as the majority of the networks have more than 25 outlets. Likewise, the massive presence of trade names in most sectors of activities means that there is now by a high degree of business competitiveness in franchising in Spain.

This consolidation of the franchising system in Spain and the strong competitiveness of Spanish trade names, along with the lack of banking finance and the generalized drop in consumption due to the consequences of the economic crisis in Spain make a high percentage of them seek new markets to offset the weak economic results of the national market.

According to the data of the [12], Spanish franchising exists in 113 countries and 148 Spanish trade names operate abroad, having 15,194 outlets. Comparing these data with those of previous years, we note the continuity of the growth trend.

Theoretical frameworks show that firms tend to internationalize first to psychically close countries and then gradually move to more psychically distant ones [13]. In fact, Portugal is the first destination of Spanish franchise networks in international markets according to data from the Spanish Association of Franchisors. Therefore, the study of international expansion of Spanish franchisors to Latin American countries is of great importance.

Latin American countries have been an important part of this internationalization process of Spanish franchising firms. In fact, a total of 66 chains are operating in the Latin American market and have a total of 3,543 outlets. This is to a great extent due to the cultural link which exists between Spain and Latin American countries.

The present work seeks to contribute to the study of international franchising by introducing several factors as the market potential and size, the per capita income and the unemployment level of the countries, among others, which have not been used in the previous literature. Also, the literature review concerning the international expansion of franchising indicates the lack of works which have exclusively centered on the Latin American market. This gap in the literature, together with the expansion of Spanish trade to the Latin American market justifies our work. Furthermore, in this study we use canonical correlation, a methodology that has not been used in the previous literature of international franchising.

This specifically analyzes the factors which are external to Spanish trade names and that determine their inclination to operate in the Latin American market. The specific aims of this work are, therefore, on the one hand, to offer a general view of the current situation of Spanish franchisors in Latin American countries and, on the other hand, to analyze what the socio-economic or external factors of Spanish franchisors in this market are.

To achieve these aims, this investigation begins with an analysis of the current situation of Spanish franchisors in foreign markets, a special emphasis being given to the Latin American market. The work continues with a review of the extant literature concerning the factors which determine the selection of markets. There is then a specific study of the socio-economic factors which determine the choice of markets for the concrete case of franchisors. Within this specific section we define the different hypotheses proposed in the work. Next, we describe our methodology, defining the variables which are the aim of the study and the canonical-correlation analysis. The results attained are shown and explained in the following section. The work finishes with the conclusions and business implications, as well as the limitations and future research lines.

### Statistics concerning the expansion of Spanish franchisors in Latin American countries

The franchising system has existed for a long time in many countries and markets and has become one of the main expansion options for the businesses of many firms. The security of a tested business model is one of the main assets of this business activity. This has meant that over the last decades it has achieved spectacular levels of development in many countries.

The franchising system began in Spain at the end of the 50s with the entry of some foreign brands, mainly from France—in the personal apparel sector—and the USA—in the fast-food sector [12]. Nevertheless, it was not until the 80s that franchising really took off in Spain, becoming a successful business format via the huge entry of foreign chains and the appearance of numerous Spanish firms. Spain has thus gone from two trade names in 1960 to 47 in 1980, 750 in 2000 and 1,114 in 2015.

The evolution of franchising in Spain has been characterized by two basic aspects: the consolidation of the system in Spain and the growing expansion of many franchisors toward other markets [14]. Nowadays, franchising exists in practically all sectors of activities. This fact, along with the high percentage of trade names with more than 25 franchisees, shows that this business system has become solidly consolidated in Spain. Parallel to this consolidation, numerous trade names, convinced of their own strength and aware of the difficulties in the national market, have begun a process of expansion to other, international markets. All the experience attained throughout the years by Spanish trade names has led to the setting up of more professionalized projects which are better prepared to expand to other markets.

In spite of the Spanish national market’s prevailing economic problems, Spanish franchising has continued with its expansion to other markets. Of the 148 Spanish chains which operate in some part of the world, 66 do so in the Latin American market. This is 44.6%, indicating the importance that this market has for Spanish franchising. Likewise, of the 15,194 Spanish outlets, 23.3% of them are located in Latin American countries.

If we analyze the chains that exist in the Latin American market (Table 1), we note that the *DIA* chain is the one which has the most franchised outlets in Latin America—856 units—followed by *MRW*, which has 601 units. Next are *Telepizza*, which has 344 units, and *No+Vello* with 343. The rest are far behind. A significant aspect instead of a massive presence of franchising in a broad variety of activities, it is restricted to a few sectors. The data in Table 2 corroborate this.

**Table 1:** The first 20 Spanish trade names present in Latin America.

TRADE NAME	No. UNITS	SECTOR
DIA	856	Food
MRW	601	Transport services
Telepizza	344	Hotels and restaurants
No+Vello	343	Beauty and cosmetics
Pressto	236	Dry cleaners
Zara	129	Fashion
Publipan	123	Marketing and communication
Mango	76	Fashion
Adolfo Domínguez	71	Fashion
Bershka	63	Fashion
SN Servicios Normativos	54	Consulting companies
Clean&Clean	53	Dry cleaners
WomenSecret	45	Fashion
Imaginarium	43	Specialized shops
Alfa Inmobiliaria	42	Real estate and financial businesses
Pull& Bear	41	Fashion
MassimoDutti	34	Fashion
Gaes	32	Health and personal care
Naturhouse	31	Health and personal care
Oysho	31	Fashion

**Table 2:** Number of outlets per sector in Latin American countries.

N° UNITS	SECTOR
856	Food
606	Fashion
601	Transport services
403	Beauty and cosmetics
356	Hotels and restauarants
298	Dry cleaners
123	Marketing and communication
65	Health and personal care
54	Consulting companies
47	Specialized shops
42	Real estate and financial businesses
30	Recycling and consumables
29	Travel agencies
15	Decoration and household
10	Renewable energies
8	Specialized services

Table 2 shows that the food sector has the greatest number of Spanish outlets in Latin American countries, all belonging to the *DIA* brand. The transport services sector also has a significant presence in Latin American countries. However, the same happens as in the food sector: there is a sole brand–*MRW*. Fashion is the sector with the strongest presence in Latin America and has 21 franchising chains. Beauty and cosmetics is another important sector in Latin America and has 11 franchising chains. In brief, it can be noted that in spite of the significant presence of Spanish franchising in Latin American countries, it still does not operate in many sectors of activities there. This is an important market niche for many Spanish trade names which are in the process of expanding their businesses toward other markets.

**Fig 1:**
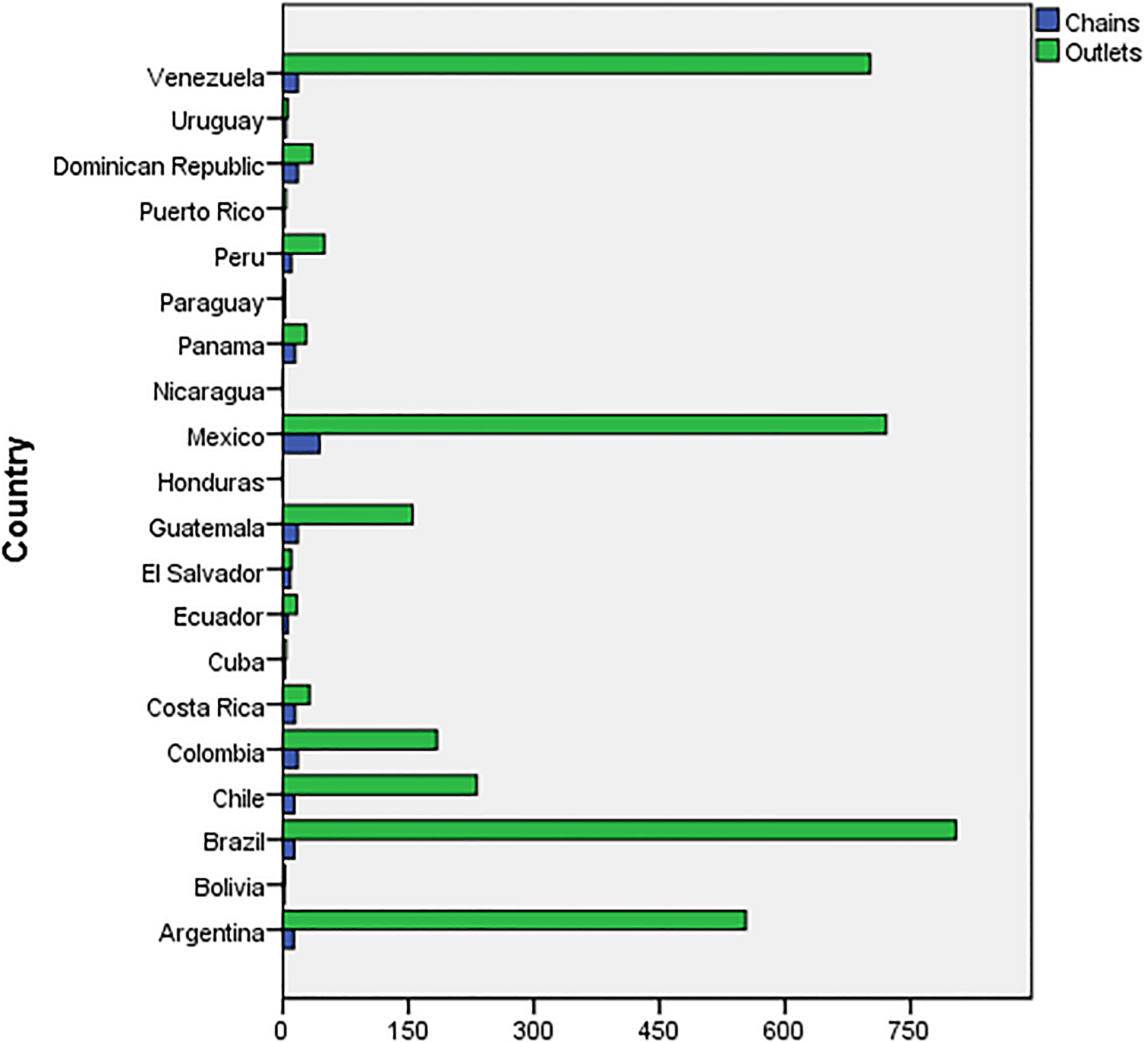
Distribution of Spanish chains and outlets in Latin American countries.

Analyzing by countries (Fig 1), it is noted that Mexico stands out from the rest as to the number of chains operating there: 19.5% of the trade names in Latin America. Then come Colombia, the Dominican Republic and Venezuela, with each having 8%. As to outlets, Brazil stands out, having 22.7% of the total of franchised units, followed by Mexico which has 20.3%, Venezuela which has 19.8% and Argentina which has 15.6%. The rest of the countries have far less. Without doubt, Brazil is one of most significant emerging markets for Spanish franchising, chiefly due to its market’s potential and maturity, its growth rate, the closeness of its culture to Spain’s and the strong development of the Brazilian franchising system [12].

In short, Spanish franchisors consider the Latin American market as one of the most important for expanding their businesses. However, despite this market’s potential, neither the number of chains nor the number of sectors of activities has attained high figures. This is why this market is a great opportunity for many Spanish chains which are currently analyzing their situation to open up toward foreign markets.

### Factors to be considered when choosing markets

Choosing their markets has become a crucially relevant fact for those firms which have decided to develop their activity in foreign markets [15, 16]. Choosing international markets has thus become a fundamental decision [17]. This decision requires information about the possible destination markets (for instance, government regulations, transaction-specific factors, global strategic variables and the dynamics and nature of market variables [18]) and the valuing of this information is going to largely determine the degree of success or failure in the international market [19]. Hence, an important aspect to take into consideration will be the analysis of the different country-markets where the firm can and must be present.

The business literature has not been oblivious to this topic and various scientific works have dwelt on this matter. There are numerous relevant studies about determining attractive markets. There is [20] a long time ago, and others which are more recent, such as [21], and [16]. All this extant literature on the subject has reached similar conclusions. Nonetheless, some differences among the studies lie in both the indicators considered and the weightings assigned to these indicators. But, generically, all converge on the need to consider indicators related with social, political, and cultural factors, amongst others, in order to establish the potential attractiveness of foreign markets. There is a broad literature in this context and different theoretical frameworks have been proposed concerning the phases in the choice of markets, the methods to use in this process and the indicators which must be employed to value the appeal of an international market [22]; among others. This work is going to center on this third approach. Despite the large number of previous studies on internationalization decisions, the academic attention paid to the expansion of franchisor to the Latin American region continues to be limited [23]. In fact, most published works merely focus on the American or British chains [24]. The aim of this work will be to find those factors which are relevant to the presence of the Spanish franchising system in the Latin American market.

Regarding the indicators for choosing markets and to explain the presence of Spanish franchisors in Latin America, models which mean to rationalize the search process for a new international market and propose criteria for choosing it began to appear in the 70s [20]. Some studies have been undertaken seeking to integrate previous works, suggesting as sets of criteria for choosing markets: the size of the product’s specific market and its growth, the availability and costs of the production factors, the level of economic development [25], and the country’s environment, the psychic distance, and the market’s competition and knowledge [26].

[27] present various types of information that exporters consider to be important when evaluating and choosing foreign markets. From the empirical research carried out among different sectors of activities, these authors conclude that all the elements analyzed coincide in placing at the top in the ranking of indicators the information on the destination country’s financial structure, as well as the information about the competition and the entry conditions. On the other hand, the information on market research, advertising and promotions, and demographic and socio-economic environments is the least valued.

A very relevant work with a theoretical framework about the choice of markets is [28] study. These authors carry out a comprehensive theoretical review of the literature on the information which can be useful when evaluating international markets. In their review they identify a total of 200 indicators, which they reduce (to achieve operability) through a process of interviews and focus groups with representatives of export-related governmental agencies, of international banking institutions and of private businesses with export experience. This process produces a theoretical framework with six dimensions that group together different indicators. Each of these dimensions and indicators coincides with the elements which the literature frequently cites as being important factors to consider when choosing a market and developing international strategies. This research also offers a ranking of the dimensions and indicators considered (Table 3) as being the most significant when selecting a market: the market’s potential, the country risk, and the cultural distance, among others.

**Table 3:** Indicators in market choice.

Dimensions	Measure
Politics	Current and future political stability, expressed by the degree of centralization of political power and the degree of representation and trust of the people in its government.
	Diplomatic relations between the foreign government and the domestic government and its effect on trade.
	The foreign government’s internal politics, attitudes and actions toward private firms.
Market Potential	Opportunities for exporters due to the current and future demand of products and services and the capability of the market to pay for these products and services.
	The market’s internal and external competition.
	Costs of adaptation associated with the products or services to be exported
Economy	Current situation of the development of the market, measured by standards of economic results.
	Strength of the market in terms of its production of products and services
	Consumption trends of products in the market.
Culture	Degree of cultural unity and national integration and the degree of ethnical and cultural differences.
	Cultural differences (distance and similarities) between the export market and the domestic market.
Infrastructure	Degree and nature of the infrastructure of the market’s physical distribution.
	Degree and nature of the market’s communication infrastructure.
	Climatological and geographical conditions.
Legal Environment	Tariffs and taxes in the market.
	Other legal considerations (laws which affect intellectual property, requirements for visas, etc.).

Table from Wood VR, Robertson KR. (2000) Evaluating International Markets: The Importance of Information by Industry, by Country of Destination and by Type of Export Transaction. International Marketing Review. 17 (1): 34–55.

### Socio-economic indicators relevant for franchisors when choosing markets

In 2012, Baena proposed that more work is necessary to identify the key factors affecting to the international expansion of franchisors [29]. In this work we are going to consider a set of variables, basically related with the destination country, although the literature indicates other factors connected with the franchisor or the franchisee that have also to be taken into account when studying the topic.

Three different types of indicators can be grouped together from this set of factors and the points cited above. Factors directly linked to the trade name are also considered (size, international experience, access to resources, experience as a franchisor, etc.). In this sense, the work of [10] determines that the international experience variables (number of years of internationally franchising), speed of internationalization (years from the origin of the franchisor to its international expansion) and the sector in which the franchisor operates are those that affect the franchisor’s strategy to compete in different country-markets simultaneously. Also, it must be noted that franchisors only use strategies of internationalization after they have accumulated local experience and knowledge [30].

A second block of factors is those related with the franchisee itself and its personal characteristics (risk aversion, individualism, financial capacity, and so on). From the work of [8] it is concluded that Spanish trade names prefer to enter markets where the franchisee has a high risk aversion (fear of failure in the business), as well as a low degree of individualism, understood as the competitive attitude of working individually vis-à-vis collectivisms [31]. This evidence notes that franchising is configured as a form of business expansion which enables the minimizing of the business-associated risks. This is why, in those markets which have people with a strong entrepreneurial character and whose risk tolerance is therefore greater (high levels of individualism), the presence of franchising chains is less [8].

A third block is those factors associated with the operation’s destination country. This is what this work analyzes. Of the different destination country factors to be taken into account in the internationalization of firms, most of the works analyzed evaluate both the cultural distance and the geographical distance. The growth of international expansion is continuing with both export and import oriented expansion being mostly within the same geographical area or those related with prior historical ties with the importing country. It seems that, all things considered, franchisors carefully consider both the geographical and cultural distance dimension [32]. These are decisive in the international markets entry mode and in the choice of markets, as their differences affect, for instance, strategic decisions [33, 34]. Nevertheless, and as [28] note, the cultural distance—considered from Hofstede’s indexes [35]- are not going to be relevant in this work; that is to say, for the internationalization of franchisors toward Latin American markets. Cultural similarities and differences in the destination countries (the Latin American market) with respect to the domestic market (Spain) as to demographic, psychographic, lifestyles and values aspects mean that this variable is not relevant. The same occurs with the geographical distance, given that this is also similar between the origin markets and the destination markets. To expand a franchise toward the Venezuelan, Colombian market or any other of that continent is very different, in terms of geographical distance, from doing so in Portugal, France or Morocco, though Hoffman et. al.'s [36] investigation concerning the internationalization of franchises results in marketing similarities seeming to present significant explanations with respect to the volume or flow of cross-border franchise activity between two nations.

Yet there is a series of variables which are going to be decisive in the choosing of destination markets and which we are going to develop.

The country risk variable, which encompasses macroeconomic aspects (inflation, depreciation the currency and interest rates) and political aspects (wars, expropriation, confiscation, currency convertibility, repatriation of incomes, tax increases, elimination of subsidies, limitations of the control and ownership of firms) has generally had little importance in the choice of the foreign markets entry mode [37, 38, 39, 40], as well as in selecting the destination markets [22, 26, 41]. Nonetheless, there seems to be a trend which indicates that firms are changing their way of valuing risks associated with the instability of political and economic conditions, mainly if the business which is being internationalized belongs to the services sector (as services firms are more workforce than capital intensive, in general, starting up activities in international markets does not tend to be accompanied by high investment, as takes place in manufacturing firms which do require much investment, mainly in installations and production technologies). Operating in international environments therefore means a series of risks related with the destination country. [40, 42, 43] propose that a greater risk favors entry modes which involve a lower commitment of resources (such as, for example, franchising). In this way, the flexibility necessary to enable the firm to adapt to the fluctuations of the external conditions is achieved without incurring high fixed costs. Notwithstanding, once franchising has been established as an entry mode, due to these associated risks those countries are chosen where the risk of failure is less. Accordingly, the hypothesis proposed is:

***Hypothesis1**. The likelihood of being considered as a destination market in the internationalization of Spanish franchising firms decreases in countries with a high Country Risk Index*.

The current and future performances of franchisor are affected by the economic criteria used in country selection [44]. A relevant factor found in the literature for choosing appropriate markets is the Market Potential. One way, among others, of measuring this is through the country’s per capita income. Studies such as [45] propose that the influence of share ownership on businesses is positively correlated with the destination’s economic development. Other studies on internationalization [37] determine that a greater economic development of the country chosen for investment favors a greater involvement of the firms’ resources commitment. However, other research [40] posits exactly the opposite. That is to say, that the markets with a greater potential are the most appropriate for using contractual modes, as according to them: a) in these markets there is greater competition and this decreases the return on the investment, b) knowledge transfer is easier and the adaptation costs less, and c) in these markets there is normally a greater legal protection in the contractual agreements [41, 46]. With respect to our study, international expansion offers firms different advantages. One of them is that through this strategy the trade names can enter the unsaturated new markets relatively quickly. This is because these markets mostly have lower levels of competition than those in the firm’s country of origin [47]. Other works have also suggested that franchising chains prefer to enter markets which have sizable growth indexes that are sustained over time. This is due to their facilitating high levels of growth and income [48]. We then propose the following hypothesis:

***Hypothesis 2**. The likelihood of a greater presence of franchisors as a business operation mode increase in countries with a high Per Capita Income*.

The market potential variable can also be defined by other economic variables, such as the unemployment rate [49, 50]. The unemployment rate is widely recognized as a key indicator of labor market performance. As an economic indicator, the unemployment rate attracts a great deal of media attention, especially during recessions and tough economic times. This factor is related to the level of economic development and it is a key criterion in emerging markets [34]. Hence, this vein suggests that there is a proportional indirect relation between the unemployment rate of the franchisee’s country of origin and the rate of expansion of franchising in that country. This is because the idea of being a franchisee becomes more attractive when the costs of the opportunity of adopting this decision decrease. In this way, the likelihood of the chain finding local partners interested in being franchisees increases and, therefore, the franchisor’s degree of internationalization in those countries is higher when the cost of the opportunity of being a franchisee decreases. Taking this argument to the extreme, those who are unemployed have a lower cost of opportunity than those who at that moment have a stable job, or who live in countries which have a solid economy that facilitates their access to a well-paid job [50]. Hence:

***Hypothesis 3**. The likelihood of a greater presence of franchisors as a business operation mode increases in countries with a high level of unemployment*.

Another of the factors which helps to understand a market’s potential is the population destiny or market size. Different studies note that the market size in terms of the population density of the investment’s destination country is a relevant factor when choosing a market [15, 17, 26]. The market size affects productivity, given that large markets allow firms to exploit scale economies. In a period of globalization, international markets have become a substitute for national markets, especially for small countries or countries whose consumption rates are dropping. The empirical evidence shows that opening up trade is positively associated with growth [27, 51, 52, 53]. There is therefore a general feeling that trade has a positive effect on growth, particularly in countries with small markets. Internationalization can hence be considered as a substitute for internal demand to determine the market size for a country’s firms. In this sense, firms seek markets with a population density which enables them to aspire to a high market potential for their products. Countries such as China, India, Brazil, etc., have become, due to their high population, the goals of many firms. Franchising firms are no exception to this reality and those firms which seek the internationalization of their trade names target markets with high population levels.

***Hypothesis 4**. The likelihood of a greater presence of franchisors as a business operation mode increases in countries with a high density of population*.

The last factor related with the destination country which is going to be considered in this work is the Global Competitiveness Index. This is established by the World Economic Forum. A country’s level of competitiveness is important because its elements are crucial for its growth, for its productivity and to boost the investment of both foreign and internal investors. A competitive country thus enables a more efficient and swift development [54]. The competitiveness of countries contributes to the factors which determine growth, help to explain why the economies of some countries are more successful than others at increasing their levels of incomes and broadens the opportunities for their populations [55]. Furthermore, this index offers business people the guidelines necessary to make investments and strategic decisions [56]. Taking these arguments as a reference for this work, the following hypothesis can be established:

***Hypothesis 5**. The likelihood of a greater presence of franchisors as a business operation mode increases in countries with a high rate of competitiveness*.

## Material and methods

To be able to verify the hypotheses proposed in this work, we used data concerning the number of Spanish franchising chains which were operating in the Latin American market in 2014 according to the Tormo & Asociados consulting company [12]. These data were obtained from a database published in the FRQ journal, edited by this company. The database had information concerning the number of Spanish franchising chains present in Latin American countries, as well as the number of outlets in each of them. From this information, the dependent variable to be used in our model is the Spanish chains’ degree of presence in Latin American countries. However, we must highlight that the use of the individual form of the two variables previously indicated can create a significant bias due to not totally reflecting the presence of Spanish franchisors in the different countries. For example, though few brands may be present in a country, they may a high number of franchised outlets. A country may also have numerous brands, yet have few franchised outlets. In such conditions it is difficult to determine what the degree or intensity of the presence of Spanish trade names in the different countries is. To avoid this problem, in this work we have used canonical-correlation analysis. Unlike multiple correlation analysis, where a single dependent variable is predicted from a set of independent variables, canonical correlation predicts multiple dependent variables from multiple independent variables. Canonical-correlation analysis is therefore applied to the study of the association between two groups of variables. One of the advantages of canonical-correlation analysis is that it does require the assumptions of multiple regression analysis, especially referring to the normality.

In canonical-correlation analysis the matrix of the data is made up of *n* rows and *p* + *q* columns, split into two sub-matrixes *X* and *Y*, of columns *p* and *q*, respectively. The *n* rows represent the observations (in this case the countries), the first *p* columns *X* are the first group’s variables (independent) and the following *q* columns *Y* are the variables of the second group or dependent variables. So, canonical-correlation analysis seeks to relate the set of independent variables *(X*_*1*_,*…*,*X*_*p*_*)*with the set of dependent variables *(Y*_*1*_,*…*,*Y*_*q*_*)*. The aim is to find pairs of variables *U = u*_*1*_*X*_*1*_*+…+u*_*p*_*X*_*p*_, *V = v*_*1*_*Y*_*1*_*+…+v*_*q*_*Y*_*q*_ with the maximum linear correlation between *U* and *V*. The variables *U* and *V* are called canonical variables. The correlation between them is known as a canonical correlation and the pair of variables (U, V) are called a canonical function.

The dependent variables to be explained in this work are the number of chains and the number of franchised outlets, while the independent or explanatory variables are the country’s risk index, its per capita income, its unemployment level, its market potential and its global competitiveness index. The data of these variables corresponding to 2014 have been obtained for each of the Latin American countries.

The data of the risk index variable have been obtained from the Organization for Economic Co-operation and Development (OECD). The OECD classifies countries taking into account various quantitative and qualitative indicators. Among the former are the record of international payments compliance and the economic and financial situations. Political risk is what is mainly valued in the qualitative aspects.

The per capita income, the unemployment level and the market size variables have been obtained from the World Bank. The World Bank defines the per capita income as the gross domestic product (GDP) divided by the population in the middle of the year. The GDP is the sum of the gross added value of all the producers resident in the economy plus all the taxes on the products, minus all the subsidies not included in the value of the products. It is calculated without making deductions for the depreciation of manufactured goods or for the depletion and degradation of natural resources. It is expressed in US dollars. The unemployment level has been measured through the unemployment rate, which is defined as the proportion of the active population without work but looking for work and available to work. Yet it must be taken into account that the definitions of active population and unemployment differ according to the country. The market size has been measured via the country’s total population.

The global competitiveness index has been obtained from the World Economic Forum (WEF). This index measures the ability of countries to provide their citizens with high levels of prosperity. In turn, this ability depends on how productively a country uses its productive resources. As a consequence, the index is a measure of the set of institutions, policies and factors which determine a country’s level of productivity.

Table 4 shows the data of each of the two models’ explanatory or independent variables in each of the Latin American countries.

**Table 4:** Explanatory variables in the Latin American countries.

COUNTRY	Country Risk	Per Capita Income (US $)	Unemployment level (%)	Market size (millions of people)	Global Competitiveness Index
Argentina	7	10,952	7.20	40.73	3.95
Bolivia	6	2,320	4.40	10.32	3.64
Brazil	3	12,576	6.00	196.94	4.28
Chile	2	14,501	7.10	17.31	4.69
Colombia	4	7,144	11.20	47.08	4.14
Costa Rica	3	8,669	7.70	4.74	4.31
Cuba	7	10,115	3.30	11.28	3.60
Ecuador	7	5,035	4.20	15.25	3.65
El Salvador	4	3,699	6.70	6.26	3.99
Guatemala	5	3,243	4.10	14.71	4.04
Honduras	6	2,277	6.30	7.78	3.89
Mexico	3	9,699	5.30	119.36	4.19
Nicaragua	7	1,632	8.00	5.91	3.57
Panama	3	8,373	4.50	3.74	4.33
Paraguay	5	3,957	5.60	6.57	3.49
Peru	3	5,970	7.80	29.62	4.11
Puerto Rico	2	26,734	15.70	3.69	4.49
Dominican Republic	5	5,486	9.90	10.15	3.72
Uruguay	3	13,724	6.00	3.38	4.23
Venezuela	7	10,728	8.30	29.5	3.48

The data were analyzed via syntax with the IBM SPSS Statistics 20software.

## Results

The first result, which is obtained by applying the syntax in the IBM SPSS software, is the multivariate of significance tests (Table 5).

**Table 5:** Significance multivariate test.

Name of the test	Approx. Value.	F hypothesis	Degrees of freedom	Error	p-value
Pillais	1.0275	2.9583	10	28	0.011
Hotellings	4.0010	4.8012	10	24	0.001
Wilks	0.1621	3.7586	10	26	0.003
Roys	0.7865				

The F statistic for Wilks’ lambda is exact.

Each of the previous tests seeks to verify if the canonical correlations is equal to 0. As each p-value is below 0.05 there is an association between the two groups of variables.

Table 6 contains the information of the eigenvalues and the canonical correlations. Each eigenvalue comes from the square of the canonical correlation between each of the variables. We observe that the first canonical correlation is 0.8868. The first canonical function is obtained in such a way that it reflects the greatest possible correlation between the two sets of variables. The first canonical function corresponds to the first eigenvalue, which explains 92.06% of the total of the correlation’s variability between the two groups of variables, while the second canonical function only explains 7.94%. Thus, the relation between both sets of variables can be determined by the first canonical function, given that it explains a higher percentage of the correlation’s variability between both sets.

**Table 6:** Eigenvalues and canonical correlations.

Root	Eigenvalue	Percentage	Accumulated percentage	Canonical correlation	Square of the correlation
1	3.6834	92.06	92.06	0.8868	0.7865
2	0.3175	7.94	100.00	0.4909	0.2410

The SPSS results also show the coefficients—both the gross coefficients and the standardized coefficients—for the dependent variables and for the independent variables. However, problems of multicollinearity make interpreting them difficult. This is why they must be explained based on the correlation between each dependent (independent) variable and the corresponding dependent (independent) canonical variable. Tables 7 and 8 show this information.

**Table 7:** Correlations between dependent and canonical variables.

Variable	Function 1	Function 2
Chains	0.3846	0.9232
Outlets	0.9529	0.3032

**Table 8:** Correlations between independent and canonical variables.

Variable	Function 1	Function 2
Country Risk Index	0.1063	-0.7524
Per capita income	0.3106	-0.0850
Unemployment level	-0.0253	-0.0750
Market Size	0.8719	0.4722
Global Competitiveness Undex	0.0236	0.6182

It can be seen that the dependent variable which is most correlated with the factor of the international presence of franchisors in the Latin American countries is the number of franchised units. Its correlation is 0.9529, while the correlation between the number of chains and the factor of the international presence of the franchisor in the Latin American countries is due more to the franchised outlets than to the number of trade names, brands or chains in these countries.

If we analyze the correlations between the independent variables and the socio-economic factors, it can be noted that the most correlated variable is the market size: 0.8719. The second most correlated value is the per capita income: 0.3106. However, the rest of the correlations are not significant, as their values are below 0.3 in absolute value. Table 9 shows a summary of the confirmation or non-confirmation of the hypotheses proposed.

**Table 9:** Hypotheses proposed, correlation and confirmation.

Hypothesis	Correlation	Confirmation
H1.A greater Country Risk Index implies less presence of Spanish franchises	0.1063	No
H2. A greater per capita income implies more presence of Spanish franchises	0.3106	Yes
H3. A greater unemployment level implies more presence of Spanish franchises	-0.0253	No
H4. A greater market potential implies more presence of Spanish franchises	0.8719	Yes
H5. A greater Global Competitiveness Index implies more presence of Spanish franchises	0.0236	No

It must be pointed out that the non-confirmation of the hypotheses means that there is no significant relation between the socio-economic factors and the presence of Spanish franchisors in Latin American countries. Thus, for example, the non-confirmation of hypothesis H1 does not imply that there is a greater presence of Spanish franchisors in the countries with a greater Country Risk Index, but that there is no significant relation between the Country Risk Index and the presence of Spanish franchisors in the Latin American countries. This is likewise the case with hypotheses H3 and H5.

## Conclusions

The previous results enable the deduction of a series of considerations concerning the socio-economic factors which determine the presence of Spanish franchisors in the Latin American market.

As has been seen in this study, internationalization through the franchising system is a recognized and relevant fact, and Spanish franchisors are increasingly more present in different markets. The brand “Spain” is gaining in importance in the world and although everything “Spanish” has always been held in high esteem in Latin American countries, the strength of this brand image only confirms this fact. In short, a strong growth has taken place in recent years both in the number of different trade names and in the number of outlets, being present in all Latin American countries.

As well as showing the presence of Spanish franchisors in these markets, the aim of this work has been to determine the relevant socio-economic factors in choosing markets. To do so, we carried out an analysis of the extant literature concerning the decisive factors in choosing markets and we have considered these five factors as being the most relevant (related with the destination market): Country Risk, Per Capita Income, Unemployment Level, Market Size and Global Competitiveness.

From the study proposed it is deduced that the Spanish trade names which expand their businesses toward the Latin American market fundamentally base their decision on two socio-economic factors: firstly, on the destination market’s size and, secondly, on the destination country’s per capita income. Therefore, Spanish franchisors do seem to consider those markets which have a large market size as to the density of population and a considerable per capita income that enable them to attain high sales indexes. Most Latin American countries have significant growth rates. Brazil is the fifth world power in growth in terms of GDP, having had growth levels of around 8 to 9% in the last three years. Other countries on the continent have growth levels of around 5%. This means that the per capita income has increased and that consumption has been activated. Therefore, Spanish franchisors have wished to be present there. Countries such as China or India have been the destination target of many firms in recent years. This is due to their growth in terms of both GDP and population size. The same is happening for some Latin American countries such as Brazil, which has approximately 300 million inhabitants. A large population and a considerable per capita income are therefore relevant factors in firms’ choice of markets.

On the other hand, it is inferred from this study that franchisors do not take into account other factors such as the destination’s Country Risk Index, its Unemployment Level or its Global Competitiveness Index. This may all be due to the franchising model itself as an entry mode abroad. This entry mode has an associated lower business risk in the event of failure, as the franchising firm’s levels of investment are relatively low. It is the franchisee who, by investing the capital, runs a greater risk. Hence, although the destination country which the firm wishes to opt for has a high Risk Index, the firm can consider it as a market target. This is because the company’s financial risk levels are low, while it gains in renown and image for being present there. As to the Unemployment Level, in countries where these levels are high, these unemployed people might see self-employment as a way out of this situation. Franchising hence may be considered as a good formula to do so. However, the Latin American culture itself and studies of entrepreneurship levels do not confirm this hypothesis. The other hypothesis not upheld is related with the levels of competitiveness. It appears that the very complexity of confirming the measurement—it is an index made up of twelve different pillars which cover a broad range of aspects—makes it not objective and direct enough to be measured and considered.

To sum up, those Spanish firms which want to operate in the Latin American market should seek countries in which the market potential or size stands out among the socio-economic factors and there is a high per capita income.

This work shows the socio-economic factors which Spanish trade names take into account when internationalizing their operations in the Latin American market. The results of this study show a series of socio-economic factors which influence the final choice of the destination country. Nevertheless, this decision is not only based on the socio-economic aspects of the destination country but also on the structure of the franchising firm itself and on the export experience toward other markets. Therefore, this study serves as a complement to other studies and helps franchisors with the difficult decision of choosing a destination for their internationalization.

### Limitations

In spite of the importance of the topic analyzed, this work has a series of limitations. One refers to the geographical area to which it is restricted. A global study—not only centered on the Latin American market—could be useful to shed more light on the internationalization of franchising firms. This is both a limitation and a future research line. The methodology used is another limitation. The use of canonical-correlation analysis enables solving the multicollinearity problem which exists in a multiple linear regression model. Nonetheless, many authors consider that it is important to have a high sample size to be able to apply this type of analysis. In this case, only the data of Latin American countries have been taken into account, which is why the sample size has been restricted to 20 units. This is a significant limitation.

### Future research lines

Regarding future research lines, it must be stressed that this work has analyzed a series of important socio-economic factors of Latin American countries to determine their influence in the internationalization of Spanish franchisors there. Despite this, it would be interesting to study other factors inherent to countries, apart from than those analyzed in this research. Thus, for example, an important factor could be the study of the level of maturity or consolidation of the franchising system in each country. That is to say, an interesting idea would be to study if the level of consolidation of franchising in Latin American countries affects the decision of Spanish franchisors to internationalize toward these countries. On the other hand, it would also be interesting to study how the internal variables of Spanish franchisors (size of the franchisor, years of franchising, sector, etc.) affect the choice of Latin American countries as destinations for their internationalization strategy. Finally, as has been emphasized before, it would also be interesting to replicate this study with markets, other than those of Latin America, to analyze the possible differences which may exist among them.

## Supporting information

S1 Dataset (XLSX)

S2 Dataset (XLSX)
